# Supplementary material for: Genotype–phenotype analysis of hearing function in patients with DFNB1A caused by the c.-23+1G>A splice site variant of the GJB2 gene (Cx26)
Source: PLoS One. 2024 Oct 22;19(10):e0309439. doi: 10.1371/journal.pone.0309439 (PMC11495561; doi:10.1371/journal.pone.0309439)
Supplement: Attachment — Submitted filename: Reviewer Attachments.docx [file pone.0309439.s002.docx]

In this study, author present the audiological features of DFNB1A on a large cohort of 134 patients with the homozygous splice site variant c.-23+1G>A , compared with 34 patients with other biallelic GJB2-genotypes. While the topic is interesting and could help to better understanding genotype-phenotype analysis of hearing function in patients with DFNB1A caused by mutations in GJB2 gene, crucial details of how the study was conducted and individuals selected are missing from the methods section.

1 The methods and study details are very vague, there are no details of how the hearing impaired patients were recruited into the study, what was the inclusion/exclusion criteria. How were the final numbers in each cohort established. Was this an observational cohort study or was there specific testing and sample collection a key time points? What was the calendar time for the study?

2 Line 119, ‘the following *GJB2*-genotypes’- the level of detail can be reduced as the information is also provided in a table.

3 Line 196-198, why did the author not choose values of hearing thresholds in normal hearing individuals as a reference

4 Line 217, ‘non-truncating (NT/NT anf TT/NT) GJB2-genotypes (n=34)’. Please verify whether there are any writing errors in ‘NT/NT anf TT/NT’.

5 Conclusions. Line 413, authors please describe the conclusions in refined language

6 English needs to be improved. Many sentences are very wordy and imprecise.
