# Supplementary material for: Genotype–phenotype analysis of hearing function in patients with DFNB1A caused by the c.-23+1G>A splice site variant of the GJB2 gene (Cx26)
Source: PLoS One. 2024 Oct 22;19(10):e0309439. doi: 10.1371/journal.pone.0309439 (PMC11495561; doi:10.1371/journal.pone.0309439)
Supplement: Attachment — Submitted filename: R2 Response to reviewers.docx [file pone.0309439.s004.docx]

**Author’s response**

The authors are sincerely grateful to the reviewers for the thorough review of our manuscript “Genotype-phenotype analysis of hearing function in patients with DFNB1A caused by the c.-23+1G>A splice site variant in the *GJB2* gene (Cx26)” (PONE-D-24-16320R2). We appreciate the dedicated time and effort for providing valuable feedback on our manuscript. All additional comments have been addressed, with corresponding changes made directly to the manuscript where appropriate. We have highlighted the changes within the manuscript. The revisions have been approved by all authors. Here is a point-by-point response to the reviewers’ comments and concerns.

**Comments to the Author**

**Reviewer #1**: The authors have addressed most of my previous comments. However, there are still a few questions that are not clear, and need to be addressed:

**1 Line 197, “PTA0.5,1.0,2.0,4.0 kHz hearing thresholds in patients with 10 different GJB2 genotypes”, the number of cases in some groups in Figure 2 is relatively small. The authors are requested to introduce the statistical method used in this section. Please provide a detailed description of the statistical methods employed in the "Statistical analysis" section at Line 172 or in the "Figure legend" section.**

**Author’s response:** We added the full description of the statistical analysis in the materials and methods section (Lines 173-187, P.7-8):

“Statistical analysis of the clinical and audiological data in patients with the c.[-23+1G>A];[-23+1G>A] genotype was performed using the Sampling program, kindly provided by M. Macaulay and adapted by M. Metspalu. Differences of the credible interval at the 95% significance level were considered statistically significant. Comparison of hearing thresholds in PTA_0.5,1.0,2.0,4.0 kHz_ of the reference group c.[-23+1G>A];[-23+1G>A] with other GJB2 genotypes was performed with a Mann–Whitney U test using by software STATISTICA version 8.0 (StatSoft Inc, USA). Differences were considered statistically significant at p<0.05. Correlation analysis of hearing thresholds in PTA_0.5,1.0,2.0,4.0 kHz_ with age in patients with c.[-23+1G>A];[-23+1G>A] genotype was performed with a r-linear regression analysis using by STATISTICA version 8.0 (StatSoft Inc, USA). Differences were considered statistically significant at p<0.05. Statistical analysis of the hearing thresholds between male and female patients with c.[-23+1G>A];[-23+1G>A] genotype was performed with a Student’s t-test using by software STATISTICA version 8.0 (StatSoft Inc, USA). Differences were considered statistically significant at p<0.05”.

**2 In the discussion section, it is recommended that the authors add a discussion on the limitations of this study.**

**Author’s response:** We added the limitations of the study (Lines 429-438, P.18).

“This genotype-phenotype study of the hearing function in patients with biallelic GJB2 pathogenic variants have a some limitations related with the focusing on the homozygous patients with rare in the world splice site variant c.-23+1G>A, which have a specific audiological features. However, a some common genotype-phenotype findings, such as symmetry, progression and gender differences of the HL may be a typycal for patients with some other GJB2 variants with mild or moderate pathological effect on hearing function. We hope that this study about hearing function in patients with c.-23+1G>A splice site variant in the GJB2 gene will be a challenge for other researchers for clearly analyzed these audiological findings in the future studies”.

Sincerely yours,

**Corresponding author Nikolay A. Barashkov**

Head of Laboratory of Molecular Genetics

Yakut Scientific Centre of Complex Medical Problems

[barashkov2004@mail.ru](mailto:barashkov2004@mail.ru)
